# Carryover effects and climatic conditions influence the postfledging survival of greater sage-grouse

**DOI:** 10.1002/ece3.1139

**Published:** 2014-11-12

**Authors:** Erik J Blomberg, James S Sedinger, Daniel Gibson, Peter S Coates, Michael L Casazza

**Affiliations:** 1 Department of Wildlife, Fisheries, and Conservation Biology, University of Maine5755 Nutting Hall, Room 210, Orono, Maine, 04469; 2 Department of Natural Resources and Environmental Science, University of NevadaReno. Mail Stop 186, Reno, Nevada, 89557; 3 Program in Ecology, Evolution and Conservation Biology, University of NevadaReno. Mail Stop 314, Reno, Nevada, 89557; 4 U. S. Geological Survey, Western Ecological Research CenterDixon Field Station, 800 Business Park Drive Suite D, Dixon, California, 95620

**Keywords:** Body condition, climate change, crossseasonal effects, life history, postfledging, recruitment

## Abstract

Prebreeding survival is an important life history component that affects both parental fitness and population persistence. In birds, prebreeding can be separated into pre- and postfledging periods; carryover effects from the prefledging period may influence postfledging survival. We investigated effects of body condition at fledging, and climatic variation, on postfledging survival of radio-marked greater sage-grouse (*Centrocercus urophasianus*) in the Great Basin Desert of the western United States. We hypothesized that body condition would influence postfledging survival as a carryover effect from the prefledging period, and we predicted that climatic variation may mediate this carryover effect or, alternatively, would act directly on survival during the postfledging period. Individual body condition had a strong positive effect on postfledging survival of juvenile females, suggesting carryover effects from the prefledging period. Females in the upper 25th percentile of body condition scores had a postfledging survival probability more than twice that (Φ = 0.51 ± 0.06 SE) of females in the bottom 25th percentile (Φ = 0.21 ± 0.05 SE). A similar effect could not be detected for males. We also found evidence for temperature and precipitation effects on monthly survival rates of both sexes. After controlling for site-level variation, postfledging survival was nearly twice as great following the coolest and wettest growing season (Φ = 0.77 ± 0.05 SE) compared with the hottest and driest growing season (Φ = 0.39 ± 0.05 SE). We found no relationships between individual body condition and temperature or precipitation, suggesting that carryover effects operated independently of background climatic variation. The temperature and precipitation effects we observed likely produced a direct effect on mortality risk during the postfledging period. Conservation actions that focus on improving prefledging habitat for sage-grouse may have indirect benefits to survival during postfledging, due to carryover effects between the two life phases.

## Introduction

The survival of young from independence to first breeding has a profound influence on both parental fitness and population persistence (Gaillard et al. 2000; Etterson et al. 2011; Nicolai and Sedinger 2012; Dybala et al. 2013). Survival during this life stage is generally lower and more variable than survival during adulthood (Clutton-Brock et al. 1987; Owen and Black 1989; Martin 1995; Van der Jeugd and Larsson 1998; Ward et al. 2004) and is also inherently more sensitive to environmental variation and anthropogenic impacts (Robinson et al. 2007; Reid et al. 2003). In avian species, prebreeding survival can be divided into two components: the interval between hatching and independence (prefledging) and between independence and first breeding (postfledging; Etterson et al. 2011). Although the prefledging period has been the subject of substantial research in many species, logistic difficulties often limit monitoring of individuals during postfledging (Hannon & Martin 2006; Vitz and Rodewald 2011). Accordingly, many of the mechanisms affecting postfledging survival have traditionally been enigmatic (Anders et al. 1997; Vitz and Rodewald 2011), despite the important role that postfledging survival often plays in avian population growth (Todd et al. 2003; Hannon & Martin 2006).

Conditions experienced during one biological period often influence the performance (i.e., survival or reproductive output) of individuals during a subsequent period. Indeed, such carryover effects are commonly observed in studies of animal ecology (see review by Harrison et al. 2011). Perhaps the most commonly cited carryover effect is that of winter territory quality in the tropics affecting both the timing of migration and ultimate reproductive performance of migrant songbirds breeding in temperate forests (e.g., Marra et al. 1998; Norris et al. 2004). Because carryover effects provide a linkage between environmental conditions and components of fitness, they represent an important concept in the evolution of avian life histories (Stearns 1992; Harrison et al. 2011).

Postfledging survival is often correlated with individual size, mass, or condition at time of fledging, which has been demonstrated for numerous taxa including songbirds (Krementz et al. 1989; Naef-Daenzer et al. 2001; Adams et al. 2006; Vitz and Rodewald 2011), seabirds (Braasch et al. 2009), and waterfowl (Owen and Black 1989; Sedinger and Chelgren 2007; Van der Jeugd and Larsson 1998). Access to food resources in foraging environments often limits growth of young birds (e.g., Sedinger et al. 1995; Sedinger and Chelgren 2007), in which case the relationship between condition and postfledging survival is reflective of a carryover effect between the pre- and postfledging periods (Vitz and Rodewald 2011; Nicolai & Sedinger 2012). Habitat quality (Ward et al. 2004; Vitz and Rodewald 2011), density dependence (Owen and Black 1989; Sedinger et al. 1995; Winiarski et al. 2012), and/or weather (Reid et al. 2003; Robinson et al. 2007; Dybala et al. 2013) may determine the availability of food to growing birds and provide the causal agent(s) that promote carryover effects.

Future patterns of global change, including those associated with climate, are likely to affect the distribution and abundance of many species. These impacts are ultimately rooted in demographic processes (Crozier 2004; Kery et al. 2006; Robinson et al. 2007; Jenouvrier 2013). The potential response of species to climate change may be evaluated by quantifying demographic responses to short-term variation in characteristics of climate, such as weather. These predictable relationships can then be used to evaluate species' vulnerability to future climate change by integrating long-range climate projections with prospective population models (Seavy et al. 2008; Jenouvrier 2013). Such a process requires information on all life stages (Radchuk et al. 2013), which is often unavailable for certain parameters such as postfledging survival. Because young birds may be inherently more sensitive to resource availability than adults (Oro et al. 2010), climatic variation is predicted to disproportionately affect survival of young birds. Carryover effects between pre- and postfledging periods may provide a mechanism linking characteristics of climate with postfledging survival. This is particularly true if seasonal resource abundance declines and affects body condition at fledging, coincident with changing weather patterns that may be associated with climate change. By producing a biological covariance among multiple demographic parameters, carryover effects might also influence the predictive ability of prospective population models (Norris 2005). Understanding how carryover effects influence the fitness of individuals and interact with other climatic processes is therefore of direct relevance to animal conservation in a changing climate (Harrison et al. 2011).

**Figure 1:**
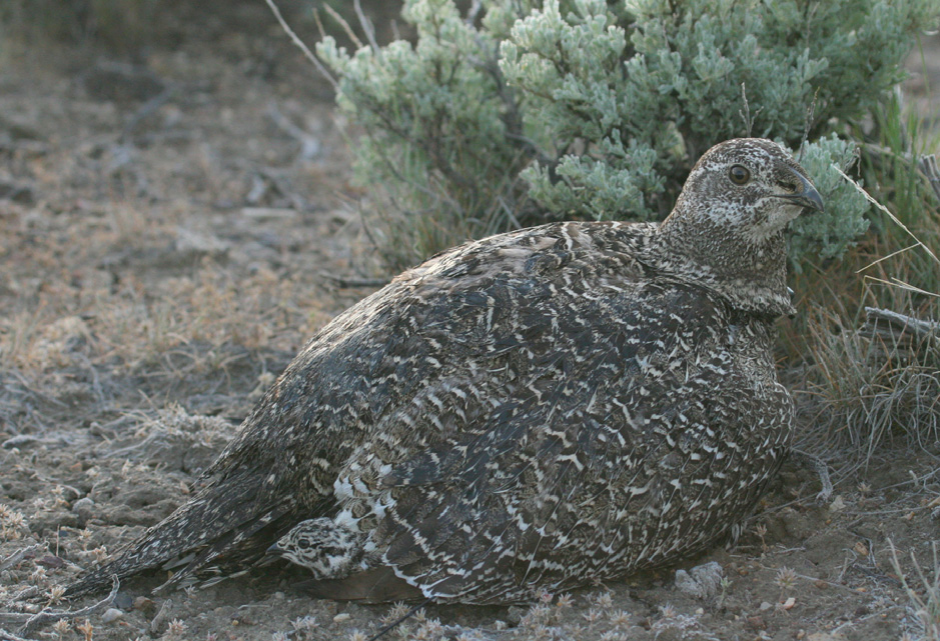
A female greater sage-grouse broods her dependent prefledging young in eastern Nevada, USA. Sage-grouse young are precocial and depend on their mother for thermoregulation during the first three to 4 weeks following hatch while developing their postnatal plumage. Following fledging, young may remain socially aggregated with their mother and brood-mates, but are no longer dependent on parental care.

We explored the effects of body condition (body mass corrected for structural size) and weather variables on postfledging survival of greater sage-grouse (*Centrocercus urophasianus*, hereafter sage-grouse; Fig.1), a species of conservation concern in western North America (Knick and Connelly 2011). In the arid and semiarid sagebrush (*Artemesia* spp.) ecosystems inhabited by sage-grouse, annual fluctuations in weather (e.g., temperature and precipitation) produce general climate patterns that are characterized by drought and nondrought periods (Miller et al. 2011). Sage-grouse vital rates respond negatively to drought conditions (Blomberg et al. 2012), and these relationships likely stem from the link between available moisture and seasonal food resources, particularly during the reproductive period (Blomberg et al. 2012, 2013c). Sage-grouse young are precocial (Schroeder et al. 1999), and females often move their flightless broods long distances (>5 km) between nesting habitats and summer brood-rearing areas (Atamian et al. 2010). Like most prefledging galliforms (Moss 1997), sage-grouse young depend on availability of invertebrates and herbaceous forbs to meet high nutritional demands associated with rapid growth (Klebenow and Gray 1968; Moss 1997; Hannon and Martin 2006), and their ability to acquire these food resources affects both their survival (Gregg and Crawford 2009; Casazza et al. 2011) and growth (Blomberg et al. 2013b). Given the wide range of conditions experienced by young sage-grouse and their high nutritional demands, the potential for prerecruitment carryover effects seems likely.

We used data collected from radio-marked juvenile (<1 year of age) sage-grouse at multiple study sites in Nevada, USA, to evaluate survival between August and the subsequent spring breeding season (March), which we defined as the postfledging period for sage-grouse. We estimated monthly and cumulative postfledging survival rates and evaluated the ecological processes that affected survival, including potential carryover effects. We hypothesized that postfledging survival would be affected by carryover effects from the prefledging period and predicted that individual body condition at fledging would be positively correlated with postfledging survival probabilities. We also hypothesized that drought conditions would negatively affect postfledging survival rates. Here, we predicted that survival would be negatively correlated with temperature and positively correlated with rates of precipitation recorded during the previous growing season; however, we considered two potential explanations for these climate–survival relationships. First, we considered that drought conditions would yield carryover effects from the prefledging period, in which case we expected body condition scores to be negatively associated with temperature and positively associated with precipitation. Alternatively, we considered that drought conditions might produce direct effects on survival during the postfledging period not related to carryover effects, in which case we expect no correlation between body condition at fledging and weather variables from the previous growing season. Because sage-grouse are sexually dimorphic and the rate of growth and size at maturity differs markedly among males and females (Schroeder et al. 1999), we considered the potential for sex-related effects in all analyses.

## Materials and Methods

### Study sites

**Figure 2:**
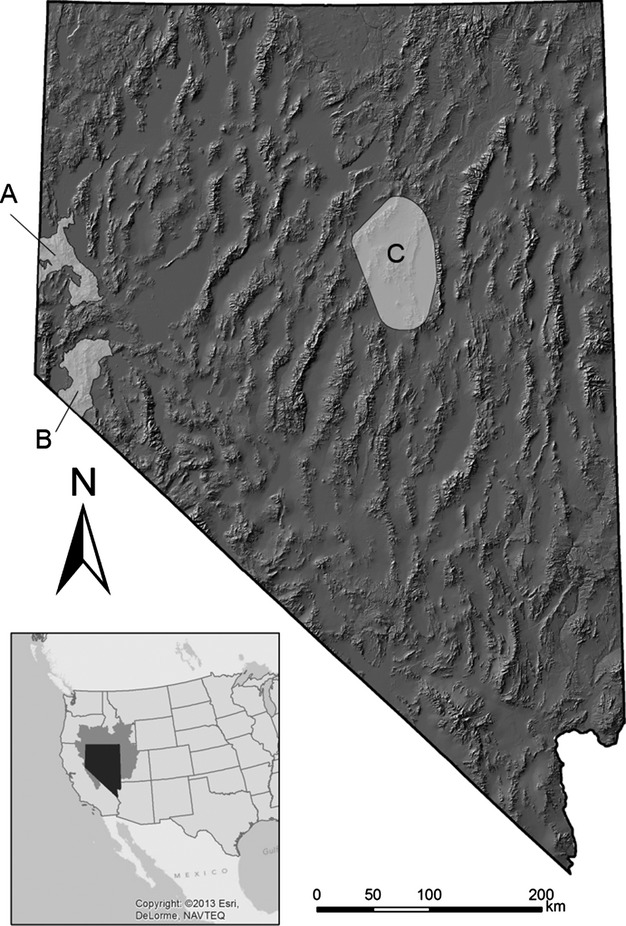
Location of study areas where juvenile sage-grouse were captured from 2006 to 2012. Study areas included the Virginia Mountains (A), Pine Nut Mountains (B), and portions of Eureka County (C). The state of Nevada (black) and Great Basin Desert floristic province (dark gray) are shown on the inset map of the western United States.

Data were collected at three study areas located throughout Nevada, USA, all of which were contained within the Great Basin Desert (Fig.2). These included Eureka County (2006–2011), the Virginia Mountains (2010–2012), and the Pine Nut Mountains (2011–2012). Sage-grouse habitat was somewhat variable among sites, but in general, vegetative communities were comprised of species typical of the sagebrush steppe and were dominated by Wyoming big sagebrush (*Artemesia tridentata wyomingensis*) or a mixed mountain big sagebrush (*A. tridentata vaseyana*)/low sagebrush (*A. arbuscula*) assemblage. Other common shrub species included common snowberry (*Symphoricarpos albus*), western serviceberry (*Amelanchier alnifolia*), bitterbrush (*Purshia tridentata*), rabbitbrush (*Chrysothamnus* spp.), and Mormon tea (*Ephedra funerea*). Conifer forests comprised of single-leaf pinyon pine (*Pinus monophylla*) and/or juniper (*Juniperus* spp.) were also present at all sites. In general, juvenile sage-grouse were found aggregated with their mothers and brood-mates in high-elevation mountain big sagebrush habitats during late summer and early fall prior to brood dispersal. Mesic site features such as wet meadows, springs, and riparian areas were selected frequently by sage-grouse during this time period (Atamian et al. 2010; Casazza et al. 2011).

### Field methods

Juvenile sage-grouse were captured by spotlighting on foot at night following protocols described by Connelly et al. (2003). We sexed sage-grouse and classified captured individuals as juveniles using feather characteristics and wing dimensions (Crunden 1963) and weighed each bird in a cloth bag using a spring balance (±50 g) or digital scale (±0.1 g). We measured tarsus length (±0.1 mm) from the intertarsal joint to the front of the foot and wing chord length (±0.1 cm) from the leading edge of the carpal joint to the tip of the longest feather. In the Pine Nut and Virginia Mountains, wing chord was measured by flattening the wing against a measurement board, whereas in Eureka County, the wing was not flattened. Additionally, in Eureka County, a single value was recorded for wing chord and tarsus, whereas in the Pine Nut and Virginia Mountains, two to three measurements were made for each bird, and we used the mean of all measurements for analysis. All birds were banded with aluminum leg rings (National Band and Tag, Newport, KY, size 14 females and size 16 males). The majority of captured sage-grouse were fitted with 22 g necklace-style radio-transmitters (model A4060; Advanced Telemetry Systems, Isanti, MN), with a small subset of birds fitted with 10.7 g transmitters at each site (model A3950; Advanced Telemetry Systems). We did not attach a radio-collar if transmitter mass exceeded 3% of body mass, and preliminary assessments suggest no relationship between transmitter size and juvenile survival (E. Blomberg, unpubl. data). Both transmitter types were equipped with a mortality sensor that doubled the signal pulse rate if the transmitter remained motionless for >8 h. Sage-grouse were monitored periodically for live/dead status throughout the fall and winter (August–February) using fixed-wing aircraft, and by ground-based field personnel during the breeding season (March–June). Because the majority of monitoring was from the air, we could not establish cause of death in most situations. All capture and handling of sage-grouse in Eureka County was approved by the University of Nevada Reno Institutional Animal Care and Use Committee (Protocol Number A05/06-22). Capture and handling of sage-grouse in the Pine Nut and Virginia Mountains was approved by the Animal Care and Use Committee, Western Ecological Research Center, U.S. Geological Survey.

### Ecological factors affecting survival

Because monitoring frequency was variable during the fall and winter, both among and within sites, we used a generalized linear modeling framework in the nest survival module of Program MARK (White and Burnham 1999) to evaluate monthly juvenile survival rates. Nest survival models are well suited to “ragged” telemetry data, where individuals are monitored at irregular intervals (Dinsmore et al. 2002; Mong and Sandercock 2007; Hupp et al. 2008). We aggregated radio-telemetry data into monthly records of live/dead status for each bird. We chose month (i.e., the calendar month) as an appropriate interval length because live/dead status was not recorded with sufficient frequency to justify intervals that were shorter. Additionally, previous studies in the Great Basin have shown that sage-grouse survival tends to vary at temporal scales that are greater than 1 month (Blomberg et al. 2013a,c). We evaluated survival from August, the earliest month during which we monitored juvenile sage grouse survival, to the following June, which represented the end of the annual nesting season (Blomberg et al. 2013c). We defined the postfledging period as the interval between the August 1 and the subsequent March 1. August 1 was chosen because at this time, juvenile sage-grouse have completed their postjuvenile moult (Johngaurd 1983) and are capable of full independence. Brood breakup typically occurs when juveniles reach 10–12 weeks of age (Schroeder et al. 1999), which in this system corresponds with late August and early September. Both sexes may continue to gain mass throughout the fall and into winter (Schroeder et al. 1999). We chose March 1 as the endpoint for the postfledging period because the onset of spring breeding occurs in March (Schroeder et al. 1999).

We evaluated a number of potential sources of variation in juvenile monthly survival using competing models and an information theoretic approach to model selection (Burnham and Anderson 2002). To examine biological processes of interest while minimizing the total number of models we considered, we employed a sequential model building procedure (Blomberg et al. 2013c). We began by evaluating temporal and spatial variation in postfledging survival associated with study site, year, and month. We first evaluated support for full annual and monthly variation in survival, as well as bimonthly variation that corresponded to the following intervals: early fall (August 1–September 30), late fall (October 1–November 31), winter (December 1–January 31), prenesting (February 1–March 31), and nesting (April 1–June 30). In the case of bimonthly variation, the resulting survival rate still reflected a monthly survival probability, but that probability was constrained to be the same between months within each two-month period. We retained the best-supported temporal structure and then considered additional models where study site was included as a categorical variable, as well as models where western Nevada sites (Pine Nut and Virginian Mountains) were allowed to differ from the eastern Nevada site (Eureka County).

Using the best-supported spatial/temporal model as a base model structure, we tested for additional individual and environmental effects on survival using individual- and group-level covariates. We were interested in evaluating differential survival among males and females and did so using a model where survival was allowed to vary by sex. We used monthly maximum temperature and total monthly precipitation data obtained for each study site from the PRISM database (PRISM Climate Group; http://prismmap.nacse.org/nn/). For precipitation, we used the total precipitation during the spring growing season (defined as 1 April to 31 July). For temperature, we used the average monthly maximum temperature during the growing season. Our primary interest for these data was to describe annual variation in drought conditions within sites. We did not evaluate spatial variation among sites with respect to temperature or precipitation, because we reasoned that our level of spatial replication (three study sites) was too low to reasonably evaluate such spatial variation. We retrieved monthly temperature and precipitation data for 10-year intervals (2002–2012) for each site, z-standardized (mean = 0, SD = 1) annual estimates within each site, and used these site-specific standardized estimates as covariate values in our analysis. This approach allowed us to model the effect of climatic variation at each site, relative to the range of variation experienced at that site over our specified 10-year interval. We considered additive effects of each covariate, as well as an interaction between temperature and precipitation, after we established that the two covariates were not strongly collinear (Pearson's *R * = * * −0.22). Severe winter weather has been shown to affect sage-grouse survival in some systems (e.g., Anthony and Willis 2009; Moynahan et al. 2006). We did not evaluate winter weather covariates, however, because winter survival is generally high for adult sage-grouse in the Great Basin (Blomberg et al. 2013c), and we recorded relatively few winter mortalities during this study.

We used the relationship between body mass and structural size as an index to individual body condition (Sedinger et al. 1995) and included these estimates as individual covariate effects. The following analyses were performed in SAS version 9.2 (SAS Institute, Cary, NC). We first conducted a principal component (PC) analysis of tarsus and wing chord lengths and extracted PC1 scores as an index to individual body size. We then used a generalized liner model to regress individual mass on PC1 scores. Because we captured individuals on differing dates among sites and years and because sage-grouse may gain mass during the fall (Schroeder et al. 1999), we also included a date term to account for variation in mass that was related strictly to date of capture. The relationship between body mass and date of capture was positive (*β * = 4.49 ± 0.67 SE) and did not deviate from a simple linear relationship. The resulting body condition estimates are therefore standardized to a common date of capture, as if all individuals were captured at fledging. For females, we conducted separate regressions for each site, because we expected site-level variance in size metrics that may be related to regional morphological variation. This also allowed us to control for the small deviations in sampling methodologies among studies (e.g., flattened vs. unflattened wing chord). For males, however, we pooled all individuals because our sample sizes were generally low (*N * = 20) and did not permit site-specific estimates. We used individual residual scores from these regressions, which can be thought of as mass relative to a common body size, as indices of body condition. Here, values >0.0 reflect individuals in above-average condition (i.e., greater amounts of fat and/or protein reserves), whereas values <0.0 reflect those individuals in below-average condition (Vitz and Rodewald 2011). We included condition score as an individual covariate in our analysis. We speculated that body condition effects may vary by sex, but our sample of juvenile males was low (*N * = 20) and insufficient to test for an interaction between sex and condition. We therefore considered two alternative model structures: one where the effect of body condition was applied equally to males and females and a second where the body condition effect was only applied to females.

Although we avoided fitting individuals with radio-collars when transmitter mass exceeded 3% of body mass, we also considered that juveniles marked early in the capture season (which were on average smaller) may have reduced survival relative to those captured later in the season because of increased risk of radio-transmitter effects. We included a date of capture covariate to explore this possibility. Finally, we considered that covariate effects may vary seasonally. For example, body condition may influence survival only during the fall when survival is generally low, but the effect may be reduced during winter when survival is typically high (Blomberg et al. 2013c). For body condition and weather variables, we evaluated models where covariate effects were applied across all months, during the fall only, and during fall and spring periods only (no winter). In all cases, we considered covariates to be supported when the following two criteria were met: (1) Inclusion of the covariate did not reduce model fit relative to similar models that did not contain the effect, based on a criteria of 2.0 ΔAIC_*c*_, and (2) when 85% confidence intervals on parameter coefficients did not overlap 0.0 (Arnold 2010). We present the estimated monthly (i.e., calendar month) survival probabilities from Program MARK. We also calculated postfledging survival, or the probability that an individual survived the entire postfledging period, as the product of all monthly survival estimates from August through February. We report these two distinct estimates as monthly survival and postfledging survival, respectively. Unless otherwise noted, survival estimates are derived from model-averaged parameter coefficients and are presented as survival probability ± SE.

Finally, to evaluate whether climatic processes affected carryover effects on postfledging survival, we compared individual body condition scores to annual site-specific temperature and precipitation values using generalized linear models in program R (http://www.r-project.org/). We contrasted a model that contained both temperature and precipitation covariates against an intercept-only null model and used similar criteria to establish importance of specific variables as described above. To establish support for our hypothesis of climatic influence on carryover effects, we set the two following criteria: (1) Body condition should have a measurable effect on postfledging survival and (2) body condition should be influenced by temperature and/or precipitation.

## Results

We captured and monitored 132 juvenile sage-grouse: 77 individuals in Eureka County between 2006 and 2011, 22 individuals in the Pine Nut Mountains during 2011 and 2012, and 33 individuals in the Virginia Mountains between 2010 and 2012. Date of capture ranged from 23 July to 6 November. The average date of capture was 4 September, and >75% of all individuals were captured during the months of August and September. The total number of individuals monitored at a single site in a given year varied from 5 to 17 (Table1). Of the 132 juvenile sage-grouse we monitored, 112 were females and 20 were males. We recorded a total of 67 mortalities across all study sites and years.

**Table 1:** Sample sizes of radio-marked juvenile sage-grouse that were monitored in three study populations in the Great Basin Desert of the United States, by site and year

Year	Eureka Co.	Pine Nut Mtn.	Virginia Mtn.
2006	18 (5)	–	–
2007	8 (2)	–	–
2008	9 (1)	–	–
2009	14 (6)	–	–
2010	17 (1)	–	7 (0)
2011	11 (0)	17 (2)	13 (3)
2012	–	5 (0)	13 (0)
Total	77 (15)	22 (2)	33 (3)

The number of male sage-grouse that were monitored for each site and year are contained in parentheses.

**Figure 3:**
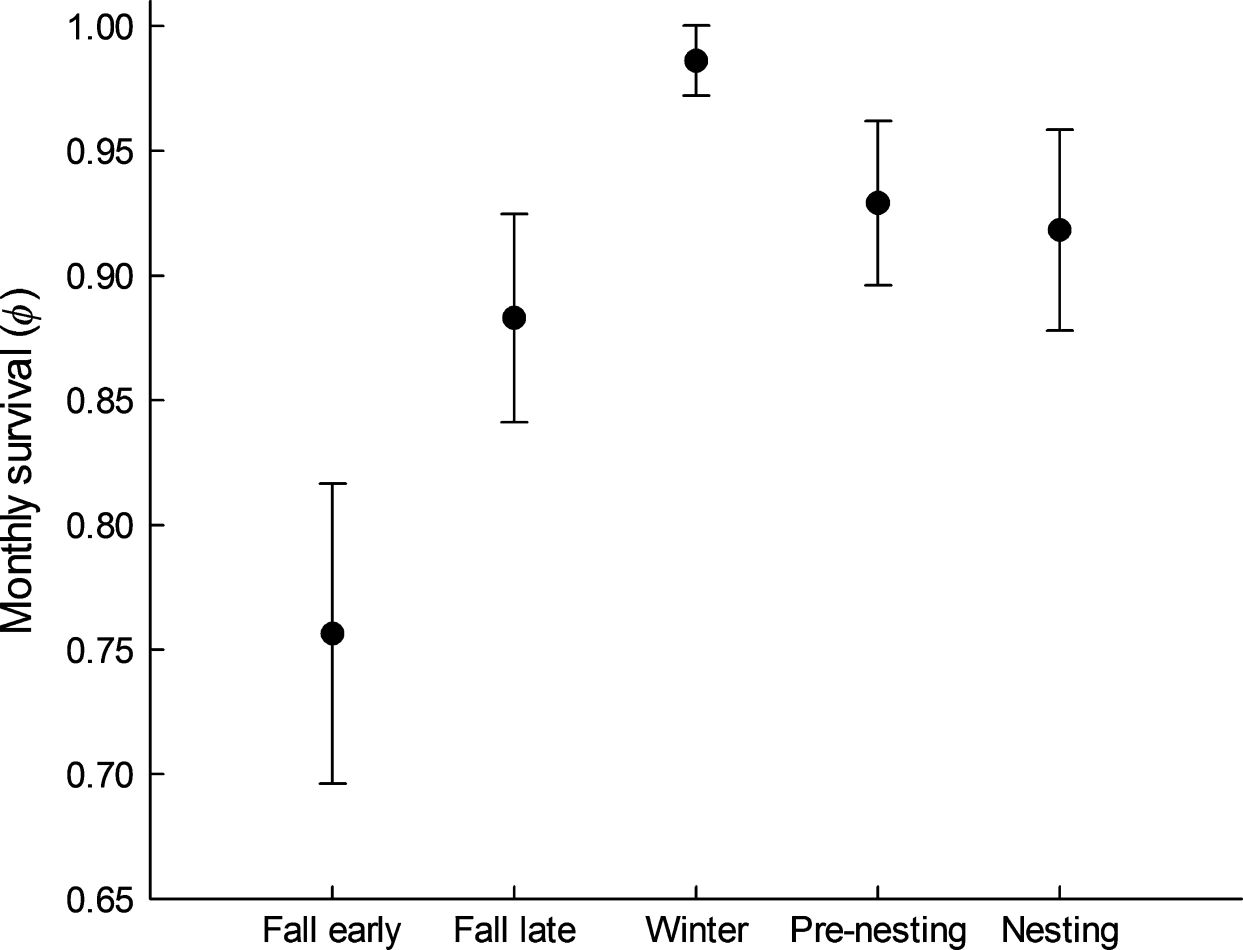
Intra-annual variation in monthly survival of juvenile sage-grouse (*N * = 136) monitored at three study sites in the Great Basin Desert of the United States from 2006 to 2012. Each data point reflects the estimated monthly survival rate (±SE), constrained to be similar between months within each of the following two-month periods: early fall (August 1–September 30), late fall (October 1–November 31), winter (December 1–January 31), prenesting (February 1–March 31), and nesting (April 1–June 30).

**Figure 4:**
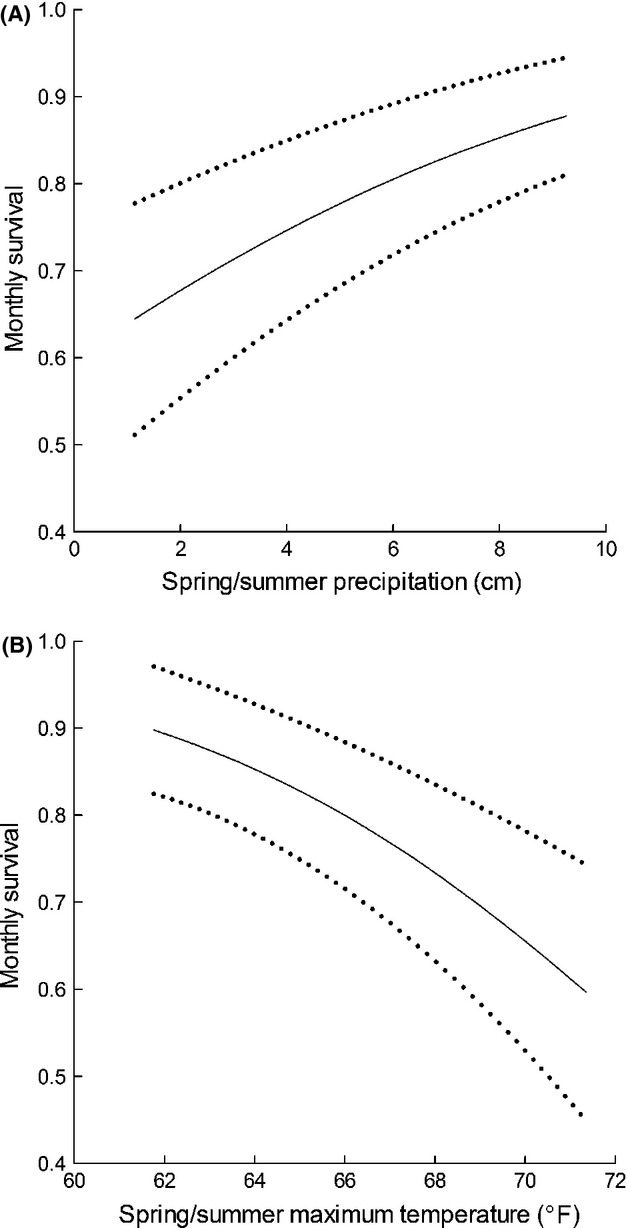
Influence of precipitation (A) and temperature (B) during the previous growing season on monthly survival of juvenile sage-grouse monitored at three study sites in the Great Basin Desert of the United States between 2006 and 2012. Total precipitation and average monthly maximum temperature were relative to the period of April 1–July 31. Dotted lines represent standard errors of modeled effects.

**Figure 5:**
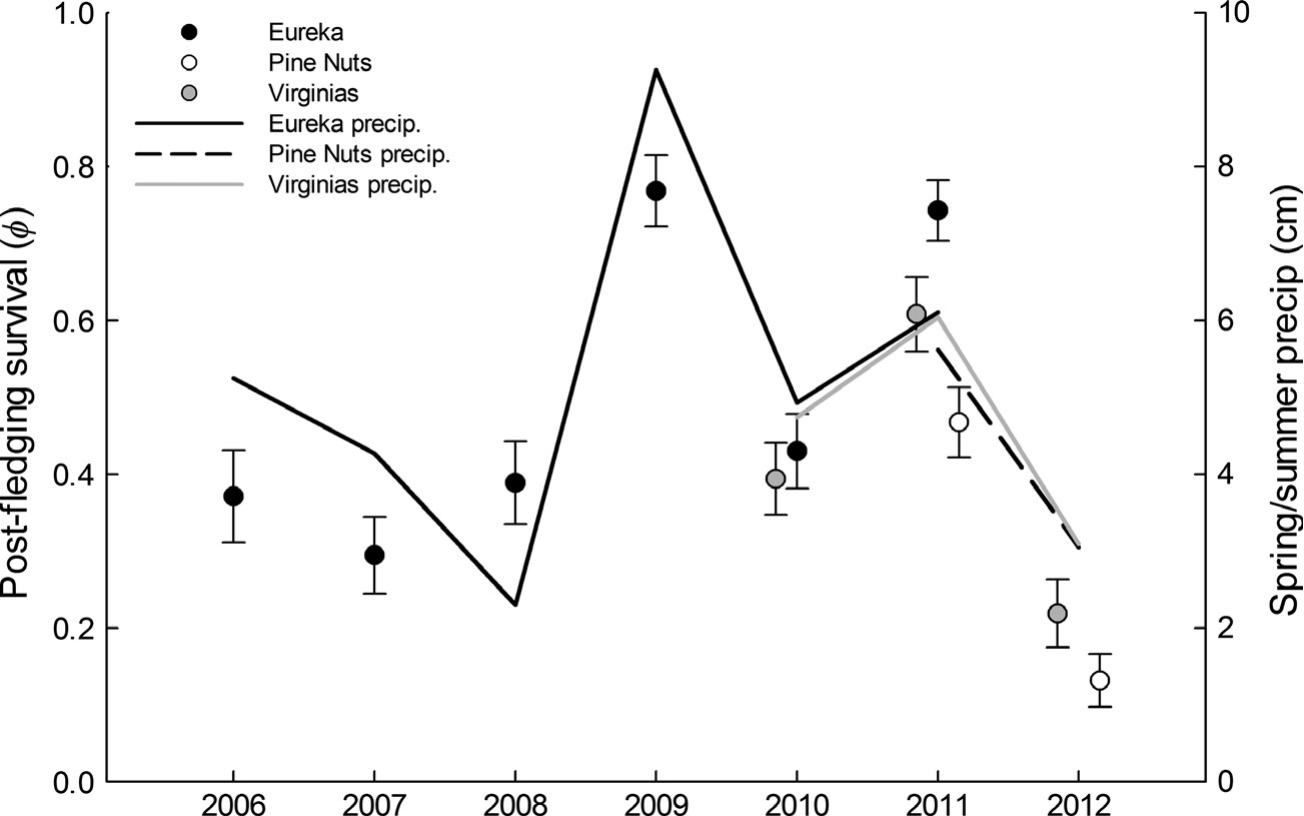
Model-averaged estimates of postfledging survival (±SE) for juvenile sage-grouse monitored at three study sites in the Great Basin Desert of the United States from 2006 to 2012. We defined the postfledging period as August 1 through March 1. Spring/summer precipitation levels by site are depicted on the secondary *y* -axis. Precipitation was relative to the period of April 1 to July 31.

Monthly survival of postfledgling juveniles was temporally dynamic, both within and among years. Within years, there was greater support for bimonthly, rather than monthly, variation in survival (Model 20 vs. Model 21; Table S1). Mean monthly survival rates were lowest during early fall (Φ = 0.76 ± 0.06), reached a peak during winter (Φ = 0.99 ± 0.01), and declined during the subsequent nesting season (Φ = 0.92 ± 0.04; Fig.3). There was also support for variation in survival among years, and this annual variation was correlated with annual variation in weather. A model containing additive effects of spring/summer precipitation and mean maximum monthly temperature (Model 11) received greater support than any other models describing interannual variation in survival (Table S1). Survival was greater in years with higher amounts of spring/summer precipitation (*β * = 0.34; 85% CI = 0.11 to 0.57) and lower maximum spring/summer temperatures (*β * = −0.45; 85% CI = −0.69 to −0.20). A two-degree increase in maximum temperature during the growing season was predicted to reduce monthly survival probability by 0.07, whereas a two-centimeter increase in precipitation during the same interval was predicted to increase monthly survival by 0.05 (Fig4). These effects were such that survival during the postfledging period varied by greater than a factor of two between years of observed precipitation and temperature extremes (Fig.5). We found evidence for spatial variation in postfledging survival that suggested juvenile sage-grouse at the eastern Nevada site (Eureka County) had higher average monthly survival (*β * = 0.89; 85% CI = 0.43–1.35) than juveniles at the western Nevada Sites (Pine Nut and Virginia Mountains).

Postfledging survival was positively influenced by an individual's body condition at fledging, and support for this effect was greatest for females only. Applying the condition effect on only the female segment of the sample (Model 1) improved model fit by 5.2 ΔAIC_c_ relative to a similar model where the condition covariate was applied equally across both sexes (Model 10; Table S1). Parameter coefficients suggested that females with larger condition scores survived at a greater rate during the postfledging period (*β * = 0.48; 85% CI = 0.22–0.74). This effect appeared to carry over throughout the postfledging period and into the following spring breeding season, but there was some suggestion that the effect was minimal during winter. A model where the body condition effect was only applied during fall and nesting periods, but not during the winter (Model 2), was competitive (ΔAIC_*c*_ = 0.25) with a model where the effect was applied equally across seasons (Model 1; Table S1). All other models that contained period-specific (e.g., fall only) body condition effects received little support (Table S1).

**Figure 6:**
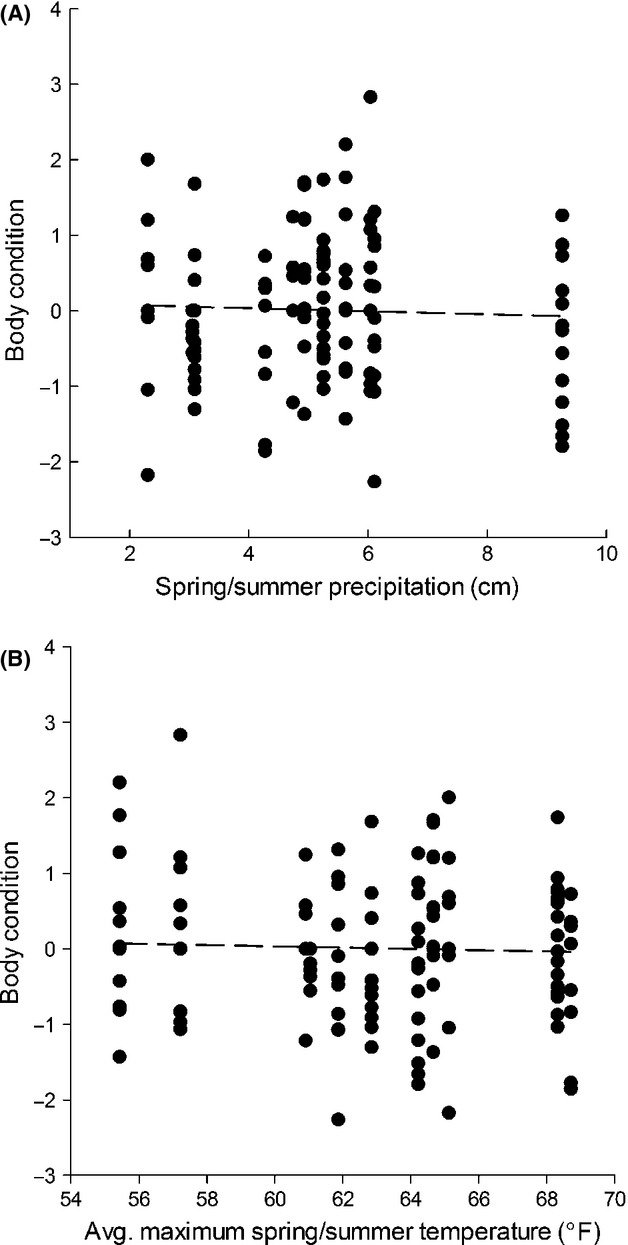
Relationship between precipitation (A) and temperature (B) during the previous growing season, and body condition at capture of juvenile sage-grouse chicks captured at three study sites in the Great Basin Desert of the United States between 2006 and 2012. The body condition index was derived from the residuals of a regression relating structural size measures to body mass.

Body condition was not influenced by precipitation (*β * = −0.03; 85% CI = −0.10 to 0.10) or temperature (*β * = −0.05; 85% CI = −0.18 to 0.08) during the prior growing season (Fig.6), suggesting that the relationship we observed between body condition and survival was independent of background climatic variation. We found little support for an effect of sex (*β * = 0.41; 85% CI = −0.20 to 1.02) or date of capture (*β * = 0.05; 85% CI = −0.16 to 0.26) on monthly survival (Table S1).

Model-averaged estimates of postfledging survival (Table2) varied from a low of 0.13 (±0.03) for the Pine Nut Mountains in 2011 to a high of 0.77 (±0.05) in Eureka County in 2009 (Table2, Fig.5). We also provide survival estimates that incorporate full temporal and spatial variation (Table2) for comparison with similar estimates reported in other studies (Taylor et al. 2012).

**Table 2:** Site- and year-specific estimates of postfledging survival of juvenile greater sage-grouse in the Great Basin Desert of the United States. We provide model-averaged estimates as well as estimates from a model where no constraints (covariate effects) were applied. The postfledging period was defined as August 1 to March 1

Site	Year	Φ_model-avg._^1^	SE	Φ_full_^2^	SE
Eureka	2006	0.37	0.06	0.43	0.06
Eureka	2007	0.29	0.05	0.37	0.09
Eureka	2008	0.39	0.05	0.32	0.07
Eureka	2009	0.77	0.05	0.76	0.05
Eureka	2010	0.43	0.05	0.71	0.06
Eureka	2011	0.74	0.04	0.58	0.06
Pine Nuts	2011	0.47	0.05	0.43	0.06
Pine Nuts	2012	0.13	0.03	0.17	0.08
Virginias	2010	0.39	0.05	0.55	0.06
Virginias	2011	0.61	0.05	0.39	0.07
Virginias	2012	0.22	0.04	0.14	0.10

1 Survival estimates derived using model averaging across all candidate models.

2 Survival estimates derived from a model that allowed full annual- and site-level variation, as well as within-year bimonthly variation.

## Discussion

We hypothesized that individual variation in postfledging survival would be influenced by carryover effects from the prefledging period. Consistent with this hypothesis, we found that body condition of females had a positive effect on their postfledging survival. When we controlled for sources of annual and spatial variation, a female in the upper 25th percentile of body conditions scores had a postfledging survival probability greater than twice that (Φ = 0.51 ± 0.06 SE) of females in the bottom 25th percentile (Φ = 0.21 ± 0.05 SE). These results are consistent with studies of other avian taxa such as waterfowl (e.g., Van der Jeugd and Larsson 1998; Sedinger and Chelgren 2007) and songbirds (e.g., Naef-Daenzer et al. 2001; Vitz and Rodewald 2011), where relationships between condition and postfledging survival were also apparent.

It is plausible that habitat-mediated availability of food resources influenced the size and mass of sage-grouse chicks at fledging and produced the carryover effects we observed during this study. Growth of prefledging sage-grouse is affected by their diet (Blomberg et al. 2013b). Specifically, individuals that consume larger amounts of invertebrate foods at an early age, and then rapidly transition to greater reliance on plant-based foods, grow to the largest size during their first month of life (Blomberg et al. 2013b). Habitats occupied during brood rearing affect the availability of these foods to young sage-grouse and are also correlated with the survival of young during the prefledging period at both local (Gregg and Crawford 2009; Casazza et al. 2011) and landscape scales (Aldridge and Boyce 2007; Atamian et al. 2010). Maternal effects on body condition may also have existed, insomuch as adult female sage-grouse make decisions regarding habitat use that influence the ultimate success of their young (Aldridge and Boyce 2007; Casazza et al. 2011). Other maternal factors, such as egg quality, inherited traits, or social dominance of the mother, may also have affected the patterns we observed (Bolton et al. 1992; Sedinger & Flint 1991; Moss 1997). However, maternal effects could only partially explain the patterns we observed, because prefledging growth is clearly linked to individual diet in sage-grouse (Blomberg et al. 2013b).

Annual variation in postfledging survival was associated with concurrent variation in temperature and precipitation, consistent with our second hypothesis of a climatic influence on postfledging survival. However, individuals that we captured during the fall were equally likely to be in good or poor condition regardless of the drought conditions they experienced during the prefledging period (Fig.6). This result may have occurred if drought conditions produced a sufficiently strong negative effect on juvenile survival during the prefledging period that a body condition threshold was required to survive to independence. Prefledging success of sage-grouse broods (Blomberg et al. 2013c) and survival of individual chicks (Guttery et al. 2013) are positively correlated with precipitation. During years of drought, individuals that could not acquire sufficient resources to maintain body condition likely failed to reach the growth threshold necessary for survival, and these individuals would have died before we could capture them at ≥3 months of age.

The residual-based methods we used to quantify individual body condition are used commonly in avian field studies (e.g., Vitz and Rodewald 2011); however, we acknowledge that support for these methods is not universal (Peig and Green 2009, 2010). In particular, these residual indices may perform poorly for comparisons of growing animals if the relationship between mass and size changes throughout growth (i.e., the index fails to account for allometric scaling during growth; Peig and Green 2010). To consider the implications of this issue for our results, we conducted a post hoc test where we calculated a scaled mass index for each individual following methods described by Peig and Green (2009) and used this as an alternative body condition covariate in our survival analysis. When we substituted the scaled mass covariate in our best-supported model, it performed more poorly but was still supported (ΔAIC_*c*_ = 1.5) and the parameter coefficient (*β * = 0.51 ± 0.25 SE) was extremely similar to our original residual-based metric (*β * = 0.48 ± 0.18 SE). These post hoc results suggest that in our specific situation, a residual-based estimate provides a reasonable approximation of individual body condition. We also acknowledge that our estimates are based on field measurements that produce a necessarily coarse index to body condition. These estimates are not without some level of sampling error. For example, we could not account for variation in digestive tract contents among individuals that would have affected the accuracy of body mass measurements. While these factors may have reduced the precision of our body condition estimates (i.e., introduced unexplained “noise”), we have no reason to suspect systematic bias among individuals related to sampling error that would have produced spurious results.

Our results demonstrate that previously established correlations between drought conditions and sage-grouse recruitment at the population level (Blomberg et al. 2012) are affected, in part, by postfledging survival. This observation also suggests that postfledging survival has an important influence on sage-grouse population dynamics in these systems. Increased frequency of drought coincident with future climate change can be expected to decrease mean annual rates of postfledging survival, thereby reducing population-level recruitment and negatively impacting sage-grouse population growth. Because carryover effects did not appear to be associated with drought conditions, there may be potential to mitigate climate change impacts through conservation measures directed at improving habitat conditions during the prefledging period. Improved quality of summer brood-rearing habitat should produce a greater number of juveniles that enter the postfledging period in higher condition and experience decreased mortality risk as a result. Conversely, negative impacts to the same brood-rearing areas are likely to result in larger net impacts at the population level than predicted based solely on direct effects on prefledging survival (Aldridge and Boyce 2007; Gregg and Crawford 2009; Atamian et al. 2010; Casazza et al. 2011). The covariance between pre- and postfledging survival rates associated with carryover effects may lead to greater population-level benefits when developing conservation strategies in general. Similarly, population modeling exercises that fail to account for the correlations between the pre- and postfledging periods are unlikely to produce accurate predictions of future population trends (Norris 2005; Kendall et al. 2011).

The seasonal patterns in survival we found are consistent with those commonly observed for grouse species in general. In a review of juvenile grouse survival worldwide, Hannon and Martin (2006) found that postfledging survival was consistently lowest during the fall and then improved overwinter. In passerines and seabirds, mortality risk is often reduced, while parents continue to provide supplemental food to their recently fledged offspring (Braasch et al. 2009; Tarwater and Brawn 2010; Dybala et al. 2013). In sage-grouse, females do not provide direct food delivery to their precocial young at any age; however, females do remain aggregated with their broods during the early fall prior to the breakup of broods when young reach 10–12 weeks of age (Schroeder et al. 1999). Although the relative level of independence of sage-grouse juveniles increases throughout the postfledging period, we found that monthly juvenile survival was lowest during early fall and reached its maximum during the winter well after juveniles had attained full independence. These patterns suggest that postfledging juveniles are not buffered against environmental variation by virtue of extended partial parental care, as has been observed in other avian systems.

In general, postfledgling survival during our study was lower than the average of previous range-wide estimates (Φ = 0.75 and 0.73 for first and renesting attempts, respectively; Taylor et al. 2012), and during our study, comparable levels of survival were only recorded in 2 years at one study site (Eureka). The estimates compiled by Taylor et al. (2012) were obtained from studies in northern and eastern portions of sage-grouse distribution, with no representative studies from the southwestern extent of the species' range. It is possible that postfledging survival of young sage-grouse tends to be lower in our region compared to elsewhere. However, few published estimates of postfledging survival exist for sage-grouse in general (e.g., Beck et al. 2006), making any conclusion regarding range-wide spatial variation in postfledging survival of sage-grouse somewhat speculative. Sage-grouse are a candidate for range-wide protection under the U.S. Endangered Species Act (United States Department of the Interior 2010) and are protected as an endangered species in Canada (Stiver 2011). Given their current status and the apparent complexities affecting postfledging survival, further investigations of this life phase are needed.

## Supporting Information

Table S1 Relative support for models of monthly juvenile sage-grouse survival at three study sites in the Great Basin Desert of the United States.
