# Supplementary material for: Carryover effects and climatic conditions influence the postfledging survival of greater sage-grouse
Source: Ecol Evol. 2014 Nov 12;4(23):4488–99. doi: 10.1002/ece3.1139 (PMC4264898; doi:10.1002/ece3.1139)
Supplement: Table S1 — Relative support for models of monthly juvenile sage-grouse survival at three study sites in the Great Basin Desert of the United States. [file ece30004-4488-sd1.docx]

Table S1. Relative support for models of monthly survival of radio-marked juvenile sage-grouse monitored at three study sites in Nevada, USA. Monthly survival was estimated using nest survival models in Program MARK (White and Burnham 1999).

|  | Model | ΔAIC_c_ | *w_i_* | *K* | Deviance |
| --- | --- | --- | --- | --- | --- |
| 1 | East/West + Temp + Precip + BiMonth + Cond (F) | 0.00 | 0.24 | 9 | 328.07 |
| 2 | East/West + Temp + Precip + BiMonth + Cond (F, No Winter) | 0.25 | 0.21 | 9 | 328.32 |
| 3 | East/West + Temp + Precip + BiMonth + Sex + Cond (F) | 1.12 | 0.14 | 10 | 327.12 |
| 4 | East/West + Temp + Precip + BiMonth + Cond (F) + Date of Capture | 1.95 | 0.09 | 10 | 327.96 |
| 5 | East/West + Temp + Precip + BiMonth + Cond (F, Fall/Spring*Winter) | 2.03 | 0.09 | 10 | 328.03 |
| 6 | East/West + Temp + Precip + BiMonth + Sex + Cond (F, Fall*Spring) | 2.27 | 0.08 | 10 | 328.27 |
| 7 | East/West + Temp + Precip + BiMonth + Sex + Cond (F, Fall Only) | 3.05 | 0.05 | 10 | 329.05 |
| 8 | East/West + Precip + BiMonth + Sex + Cond (F) | 4.04 | 0.03 | 9 | 332.10 |
| 9 | East/West + Temp + BiMonth + Sex + Cond (F) | 4.69 | 0.02 | 9 | 332.75 |
| 10 | East/West + Temp + Precip + BiMonth + Cond | 5.22 | 0.02 | 9 | 333.28 |
| 11 | East/West + Temp + Precip + BiMonth | 5.73 | 0.01 | 8 | 335.85 |
| 12 | East/West + Temp + Precip + BiMonth + Sex | 6.61 | 0.01 | 9 | 334.68 |
| 13 | East/West + Temp*Precip + BiMonth | 7.68 | 0.01 | 9 | 335.75 |
| 14 | Site + Temp + Precip + BiMonth | 7.78 | 0.00 | 9 | 335.85 |
| 15 | East/West + Temp + BiMonth | 8.39 | 0.00 | 7 | 340.55 |
| 16 | East/West + Precip + BiMonth | 10.07 | 0.00 | 7 | 342.24 |
| 17 | Site + Temp + BiMonth | 10.42 | 0.00 | 8 | 340.54 |
| 18 | Year + BiMonth | 11.73 | 0.00 | 11 | 335.67 |
| 19 | East/West + Year + BiMonth | 12.86 | 0.00 | 12 | 334.73 |
| 20 | Site + Year + BiMonth | 14.26 | 0.00 | 13 | 334.05 |
| 21 | Site + Year + Month | 16.17 | 0.00 | 17 | 327.58 |
| 22 | Site + BiMonth | 20.93 | 0.00 | 7 | 353.10 |
| 23 | Season + Year + BiMonth | 21.12 | 0.00 | 11 | 345.05 |
| 24 | Year | 32.08 | 0.00 | 7 | 364.24 |
| 25 | Site + Year | 35.33 | 0.00 | 9 | 363.40 |
| 26 | Site + Year | 35.33 | 0.00 | 9 | 363.40 |
| 27 | Site + Temp + Precip | 35.42 | 0.00 | 5 | 371.67 |
| 28 | Precip | 44.48 | 0.00 | 2 | 386.79 |
| 29 | Temp + Precip | 45.10 | 0.00 | 3 | 385.40 |
| 30 | Null | 52.80 | 0.00 | 1 | 397.13 |

^a^ Model selection notation generally follows Burnham and Anderson (2002). East/West = site-level variation between Eureka (east) and Pine Nuts/Virginia Mountains (west) study sites. Temp = average monthly maximum temperature from April – July each study year. Precip = total precipitation during April – July each study year. BiMonth = Monthly survivals constrained to be similar during the following two-month intervals: Early Fall (August 1 – September 30), Late Fall (October 1 – November 31), Winter (December 1 – January 31), Pre-Nesting (February 1 – March 31), and nesting (April 1 – June 30). Cond = Body condition index derived from the residuals of a regression of structural size on body mass. Condition effects were assessed for all birds and for females only (F), and for the entire post-fledging period, as well as for period-specific intervals (e.g. “No Winter” = fall and nesting seasons only). Date of Capture = date an individual was captured and entered the study.

**References**

Burnham, K.P., & Anderson D.R. (2002) *Model Selection and Multimodel inference: A practical information-theoretic approach*. Springer-Verlag, New York, USA.

White, G.C., & Burnham, K.P. (1999) Program MARK: survival estimation from populations of marked animals. *Bird Study*, **46**, 120-139.
